# CHA2DS2-VASc score as an independent outcome predictor in patients hospitalized with acute ischemic stroke

**DOI:** 10.1371/journal.pone.0270823

**Published:** 2022-07-13

**Authors:** Chun-Hung Su, Chien-Hsien Lo, Hsin-Hung Chen, Chin-Feng Tsai, Hei-Tung Yip, Kai-Cheng Hsu, Chung Y. Hsu, Chia-Hung Kao, Taiwan Stroke Registry Investigators

**Affiliations:** 1 Institute of Medicine, School of Medicine, Chung Shan Medical University, Taichung, Taiwan; 2 Division of Cardiology, Department of Internal Medicine, Chung-Shan Medical University Hospital, Taichung, Taiwan; 3 Division of Endocrinology and Metabolism, Department of Internal Medicine, Asia University Hospital, Taichung, Taiwan; 4 Chung Sheng Clinic, Nantou, Taiwan; 5 Department of Law, Providence University, Taichung, Taiwan; 6 Management Office for Health Data, China Medical University Hospital, Taichung, Taiwan; 7 College of Medicine, China Medical University, Taichung, Taiwan; 8 Artificial Intelligence Center for Medical Diagnosis, China Medical University, Taichung, Taiwan; 9 Department of Neurology, China Medical University Hospital, Taichung, Taiwan; 10 Graduate Institute of Biomedical Sciences, College of Medicine, China Medical University, Taichung, Taiwan; 11 Department of Nuclear Medicine and PET Center, China Medical University Hospital, Taichung, Taiwan; 12 Department of Bioinformatics and Medical Engineering, Asia University, Taichung, Taiwan; 13 Center of Augmented Intelligence in Healthcare, China Medical University Hospital, Taichung, Taiwan; Sapienza - University of Rome, ITALY

## Abstract

**Purpose:**

Atrial fibrillation (AF) is a significant independent risk factor for 1-year mortality in patients with first acute ischemic stroke (AIS). The CHA2DS2-VASc score was initially developed to assess the risk of stroke in patients with AF. Recently, this scoring system has been demonstrated to have clinical value for predicting long-term clinical outcomes in AIS but the evidence is insufficient. This large-scale prospective cohort study investigated the independent predictive value of the score in such patients.

**Methods:**

We included patients with AIS from the Taiwan Stroke Registry (TSR) during 2006–2016 as the present study population. Patients were divided into those with high (≥2) and low (<2) CHA2DS2-VASc scores. We further analyzed and classified patients according to the presence of AF. The clinical endpoint was major adverse cardiac and cerebrovascular events (MACCEs) at 1 year after the index AIS.

**Results:**

A total of 62,227 patients with AIS were enrolled. The median age was 70.3 years, and 59% of the patients were women. After confounding factors were controlled, patients with high CHA2DS2-VASc scores had significantly higher incidence of 1-year MACCEs (adjusted hazard ratio [HR] = 1.63; 95% confidence interval [CI] = 1.52, 1.76), re-stroke (adjusted HR = 1.28; 95% CI = 1.16, 1.42), and all-cause mortality (adjusted HR = 2.03; 95% CI = 1.83, 2.24) than those with low CHA2DS2-VASc scores did. In the comparison between AF and non-AF groups, the AF group had increased MACCEs (adjusted HR = 1.74; 95% CI = 1.60, 1.89), myocardial infarction (adjusted HR = 4.86; 95% CI = 2.07, 11.4), re-stroke (adjusted HR = 1.47; 95% CI = 1.26, 1.71), and all-cause mortality (adjusted HR = 1.90; 95% CI = 1.72, 2.10). The Kaplan–Meier curve revealed that both CHA2DS2-VASc scores and AF were independent risk predictors for 1-year MACCEs and mortality.

**Conclusions:**

The CHA2DS2-VASc score and AF appeared to consistently predict 1-year MACCEs of AIS patients and provide more accurate risk stratification. Therefore, increased use of the CHA2DS2-VASc score may help improve the holistic clinical assessment of AIS patients with or without AF.

## Introduction

Ischemic stroke risk is increased fivefold in patients with atrial fibrillation (AF) [1, 2]. More than half of all cardioembolic strokes are related to AF [3]. In addition, the Framingham study showed that AF is a significant independent risk factor for 1-year mortality in first acute ischemic stroke (AIS) and the elderly are particularly vulnerable to stroke when AF is present [4]. The CHA2DS2-VASc (congestive heart failure, hypertension, age ≥75 years, diabetes mellitus [DM], previous stroke, vascular disease, age 65–74 years, sex category) score is a clinical risk-stratification tool initially used to assess the risk of stroke in patients with non-valvular AF, with a score of ≥2 defined as high risk [5]. The current American Heart Association [6] and European Society of Cardiology guidelines [7] still recommend using this validated scoring system to establish the indication for oral anticoagulation therapy.

However, because all components of the CHA2DS2-VASc score are important cardiovascular risk factors, previous studies have reported the use of this scoring system in predicting clinical prognosis, including the in-hospital mortality of patients with acute coronary syndrome and long-term cardiac outcomes in older patients and those with acute myocardial infarction (AMI) [8–10]. A systematic review demonstrated that the CHA2DS2-VASc score is a useful tool for identifying AF patients at higher risk of 1–5 year all-cause mortality and that the CHA2DS2-VASc score is correlated with the development of AMI, cardiovascular hospitalization, outcome in stroke, and major adverse cardiovascular events (MACEs) [11, 12].

The CHA2DS2-VASc score has been reported to have clinical value for predicting the severity of infarction and short to long-term clinical outcomes after AIS with and without AF in addition to its original purpose of assessing the risk of stroke in patients with AF. However, the evidence is not sufficiently strong due to short-term follow-up and limited studies and the authors suggest that further studies should be conducted to modify the stratification instrument for history of AF and other concomitant risk factors [13, 14].

To date, no consensus has been reached on the usefulness of the CHA2DS2-VASc score to estimate endpoint outcomes in different clinical diseases. In addition, there is still no a clinically useful tool to predict the outcomes after AIS. Thence, this large-scale cohort study using the Taiwan Stroke Registry (TSR), aimed to investigate the independent predictive value of the CHA2DS2-VASc score in 1-year major adverse cardiac and cerebrovascular events (MACCEs) after AIS.

## Materials and methods

### Data source

The TSR is a prospective, multicenter registry of patients with acute stroke admitted to 54 major hospitals in Taiwan [15]. The aims of the TSR are to investigate the risk factors and outcomes of stroke in a nationwide registry and to assess the quality of stroke care. It includes the clinical data and outcomes of all patients with stroke. The registry was established in 2006 and involved 65 academic and community hospitals. Data includes demographic profiles, timetables of stroke onset, inpatient records, discharge information, and follow-up information. Informed consent was obtained from all patients before they were included in the registry. The research ethics committee of China Medical University Hospital in Taiwan approved the present study protocol (CMUH102-REC1-086(CR-7)).

### Ethics statement

The TSR encrypts personal information of the patients to protect privacy and provides researchers with anonymous identification numbers associated with relevant claims information, including sex, date of birth, medical services received, and prescriptions. Therefore, patient’s consent is not required to access the TSR. This study was approved to fulfill the condition for exemption by the Institutional Review Board (IRB) of China Medical University (CMUH102-REC1-086(CR-7)). The IRB also specifically waived the consent requirement. **We confirm that all methods were carried out in accordance with relevant guidelines and regulations.**

### Study population and data collection

**Fig 1:**
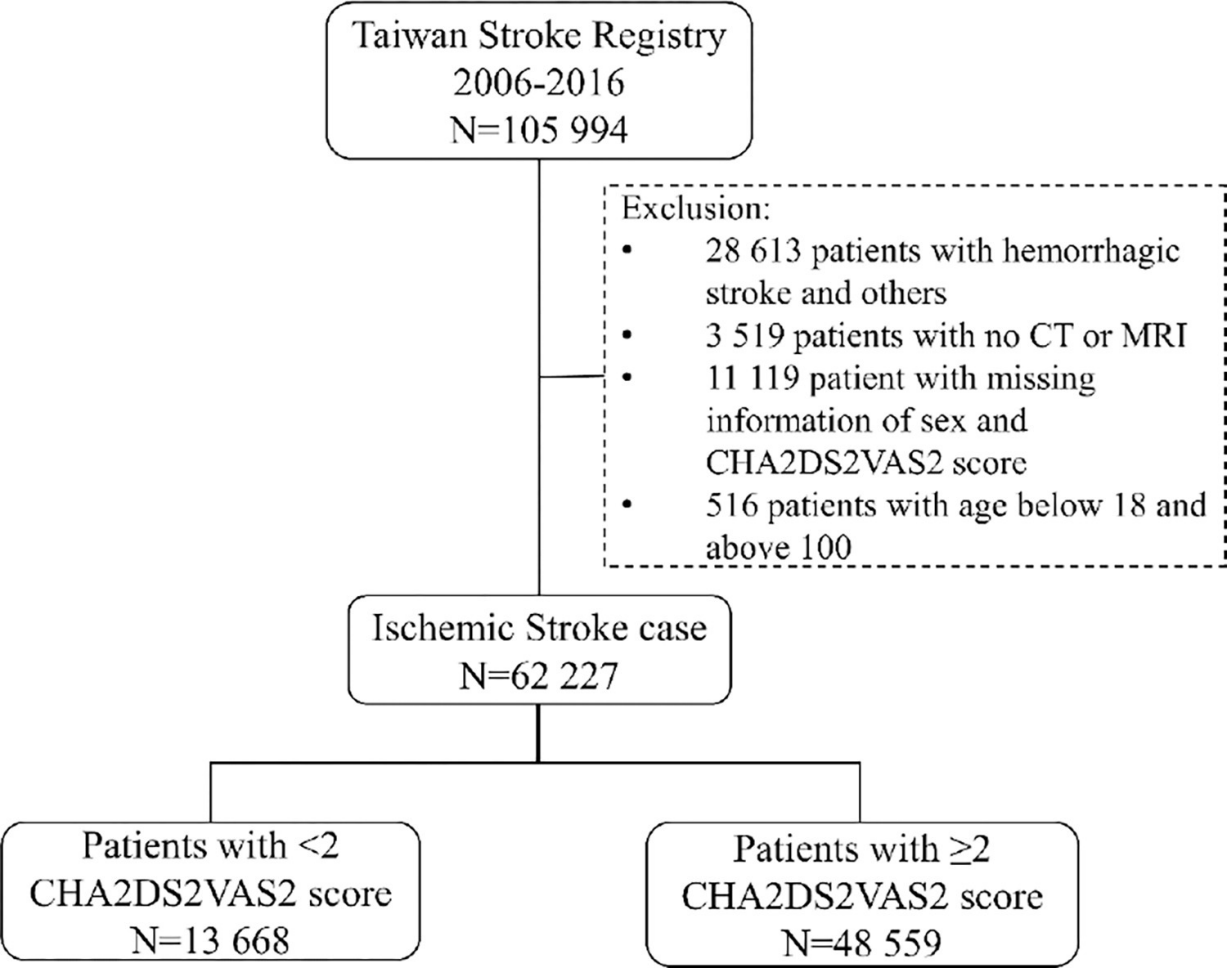
Patient flow in the study comparing high and low CHA2DS2-VASc score.

After patients with hemorrhagic stroke and transient ischemic attack (TIA) were excluded, all patients with AIS were enrolled in our study if the following inclusion criteria were met: (1) aged >18 and <100 years, (2) underwent computed tomography and/or magnetic resonance imaging for the index event, and (3) had complete CHA2DS2-VASc score data at admission. TSR registration data recorded between August 1, 2006, and August 31, 2016, were retrieved and comprised 62,227 patients who met the inclusion criteria (Fig 1). Data were prospectively collected through web-based data entry by TSR-trained study nurses. To assess the impact of CHA2DS2-VASc score and AF on stroke outcome, we collected the following data: (1) preadmission demographic profile, previous medical history of heart failure, hypertension, DM, stroke, TIA, and vascular diseases (inclusive of peripheral arterial disease and previous myocardial infarction); (2) previous AF history or new AF recorded on electrocardiography (ECG) or 24-h Holter ECG during admission; and (3) occurrence of myocardial infarction, re-stroke, or all-cause mortality at 1, 3, 6, and 12 months after the index stroke.

### Outcome measurement

The clinical endpoint was MACCE (a composite of myocardial infarction, re-stroke, or all-cause mortality) 12 months after the onset of the index AIS. We defined a CHA2DS2-VASc score of ≥2 points as high and 0–1 points as low according to its original score distinction [5]. For the cumulative incidence of endpoints and survival analysis, we divided all patients into four groups according to their CHA2DS2-VASc score and presence of AF into Group I (reference group; low score without AF), Group II (low score with AF), Group III (high score without AF), and Group IV (high score with AF).

### Statistical analysis

The difference in the distribution of continuous variables between the two groups was tested using the standard mean difference (SMD). A SMD less than 0.1 means there was no difference between two groups. Hazard ratios (HRs) were estimated using the Cox proportional model. We made two adjustments for HR: one with AF and body mass index (BMI) and the other with BMI, sex, age, heart failure, hypertension, diabetes, previous stroke, and vascular disease. A survival curve was plotted using the Kaplan–Meier method and assessed with a log-rank test. The risk ratio of each risk factor of the CHA2DS2-VASc score was calculated and visualized on a forest plot. The performance of the predictive model was evaluated by the area under the curve (AUC) of the receiver operating characteristic curve (ROC). All statistical analyses were performed using SAS version 9.4 (SAS Institute, Inc., Cary, NC, USA). A p value of <0.05 was considered significant for all analyses.

## Results

### Patient characteristics

A total of 62,227 patients (25,497 males and 36,730 females) with AIS who met the inclusion criteria were enrolled. Their median age was 70.3 years, and 59.0% of the patients were women. A total of 48,559 patients (78.0%) had high CHA2DS2-VASc scores and 4,627 patients (7.44%) had AF. In the 1-year cohort, we identified 7105 patients (11.4%) with MACCEs and 55,122 patients without MACCEs, as shown in Table 1. Patients with high CHA2DS2-VASc scores had lower BMIs (24.5 vs. 24.9) and older age (72.4 vs. 56.1) than those with low CHA2DS2-VASc scores did. Patients with high CHA2DS2-VASc scores also had significantly more comorbidities, including AF (8.5% vs. 3.8%), hypertension (86.9% vs. 48.3%), DM (50.6% vs. 8.73%), vascular diseases (6.46% vs. 14.3%) and previous stroke or TIA (1.3% vs. 0%).

**Table 1:** Characteristics and comorbidities of patients at baseline.

	CHA2DS2-VASc scores <2			CHA2DS2-VASc scores ≥2			
	N = 13 668			N = 48 559			
Variables	N	n	%	N	n	%	SMD
**Gender**							0.922
female	13 668	12 168	89.0	48 559	24 562	50.6	
male	13 668	1 500	11.0	48 559	23 997	49.4	
**Height(cm)**							0.797
mean, (±SD)	12 552	165.4	(±7.09)	43 058	159.4	(±8.39)	
**Weight(kg)**							0.419
mean, (±SD)	12 841	68.3	(±12.5)	44 470	62.3	(±12.2)	
**BMI (kg/m2)**							0.118
mean, (±SD)	12 432	24.9	(±3.91)	42 276	24.5	(±4.08)	
**Age**							1.510
mean, (±SD)	13 668	56.1	(±10.3)	48 559	72.4	(±11.2)	
**Atrial fibrillation**	13 667	524	3.8	48 553	4103	8.5	0.193
**Heart failure**	13 668	33	0.2	48 559	1458	3.0	0.220
**Hypertension**	13 668	6 598	48.3	48 559	42213	86.9	0.907
**Diabetes**	13 668	1 193	8.73	48 559	24547	50.6	1.303
**Vascular diseases**	13 668	883	6.46	48 559	6926	14.3	0.274
**Previous stroke**	13 668	0	-	48 559	661	1.4	0.166
**Previous TIA**	13 405	0	-	47 355	623	1.3	0.062
**Discharge medications**							
Anti-platelet agents	13 648	11 032	80.8	48 503	37551	77.4	0.084
Anti-HTN agents	13 564	4 921	36.3	48 385	26083	53.9	0.360
Statin	13 668	2 738	20.0	48 559	9096	18.8	0.005
**TOAST category**							
Large artery atherosclerosis	13 668	3 156	23.9	48 559	12 809	26.4	0.076
Small vessel occlusion	13 668	5 669	41.5	48 559	17 358	35.8	0.118
Cardioembolism	13 668	578	4.23	48 559	460	0.95	0.208

SD: standard deviation; BMI: body mass index; TIA: Transient ischemic attack; HTN: hypertension; TOAST: trial of ORG 10172 in acute stroke treatment)

SMD: standard mean difference (<0.1 means negligible difference between groups)

### CHA2DS2-VASc score and 1-year outcomes

Table 2 presents the association between CHA2DS2-VASc score and 1-year clinical outcomes. Among patients with high and low CHA2DS2-VASc scores, 6,143 of the 48,559 (12.7%) and 984 of the 13,668 (7.2%), respectively, reached the composite endpoints. After the confounding factors of AF and BMI were controlled, patients with high CHA2DS2-VASc scores had significantly higher incidences of 1-year MACCEs (adjusted HR = 1.63; 95% CI = 1.52, 1.76), re-stroke (adjusted HR = 1.28; 95% CI = 1.16, 1.42), and all-cause mortality (adjusted HR = 2.03; 95% CI = 1.83, 2.24) than those with low CHA2DS2-VASc scores did.

**Table 2:** The association of CHA2DS2-VASc score and 1 year outcomes.

	CHA2DS2-VASc scores											
	0–1			≥2								
Outcome	N	PY	IR	N	PY	IR	Crud HR	(95% CI)	p-value	adjusted HR^†^	(95% CI)	p-value
**MACCE**	984	6457	1.52	6143	22022	2.79	1.78	(1.67, 1.91)***	<0.001	1.63	(1.52,1.76)***	<0.001
all-cause mortality	502	6619	0.76	4057	22665	1.79	2.30	(2.10, 2.52)***	<0.001	2.03	(1.83,2.24)***	<0.001
myocardial infarction	2	6618	0.003	34	22656	0.02	4.95	(1.19, 20.6)*	0.03	3.85	(0.92,16.2)	0.07
re-stroke	510	6506	0.78	2272	22254	1.02	1.29	(1.17, 1.42)***	<0.001	1.28	(1.16,1.42)***	<0.001

N: number of event; PY: person-years; IR: incidence rate per 10 person-years; HR: hazard ratio; CI: confidence interval; MACCE: major adverse cardiac and cerebrovascular events

^†^: adjusted by body mass index and atrial fibrillation

*:p-value < 0.05

***: p-value<0.001

### AF and 1-year outcomes

The risk of MACCEs between patients with and without AF is compared in Table 3. In patients with and without AF, 845 of 4,627 (18.2%) and 6,282 of 57,600 (10.9%), respectively, reached the composite endpoints. After adjustment for all risk factors in the CHA2DS2-VASc score (sex, age, heart failure, hypertension, DM, previous stroke, and vascular disease), the AF group was associated with increased MACCEs (adjusted HR = 1.74; 95% CI = 1.60, 1.89), myocardial infarction (adjusted HR = 4.86; 95% CI = 2.07, 11.4), re-stroke (adjusted HR = 1.47; 95% CI = 1.26, 1.71), and all-cause mortality (adjusted HR = 1.90; 95% CI = 1.72, 2.10).

**Table 3:** The association of atrial fibrillation and 1 year outcomes.

	Atrial fibrillation											
	No			Yes								
Outcome	N	PY	IR	N	PY	IR	crude HR	(95% CI)	p-value	adjusted HR†	(95% CI)	p-value
**MACCE**	6282	27028	2.32	845	1448	5.84	2.17	(2.02,2.33)***	<0.001	1.74	(1.60,1.89)***	<0.001
all-cause mortality	3904	27786	1.41	655	1495	4.38	2.66	(2.44,2.89)***	<0.001	1.90	(1.72,2.10)***	<0.001
myocardial infarction	26	27778	0.01	10	1493	0.07	6.92	(3.33,14.4)***	<0.001	4.86	(2.07,11.4)***	<0.001
re-stroke	2568	27281	0.94	214	1476	1.45	1.49	(1.30,1.71)***	<0.001	1.47	(1.26,1.71)***	<0.001

N: number of event; PY: person-years; IR: incidence rate pre 10 person-years; HR: hazard ratio; CI: confidence interval; MACCE: major adverse cardiac and cerebrovascular events

^†^: adjusted by body mass index, sex, age, heart failure, hypertension, diabetes, previous stroke and vascular.

***: p-value<0.001

### Cumulative incidence of MACCE and survival curve

**Fig 2:**
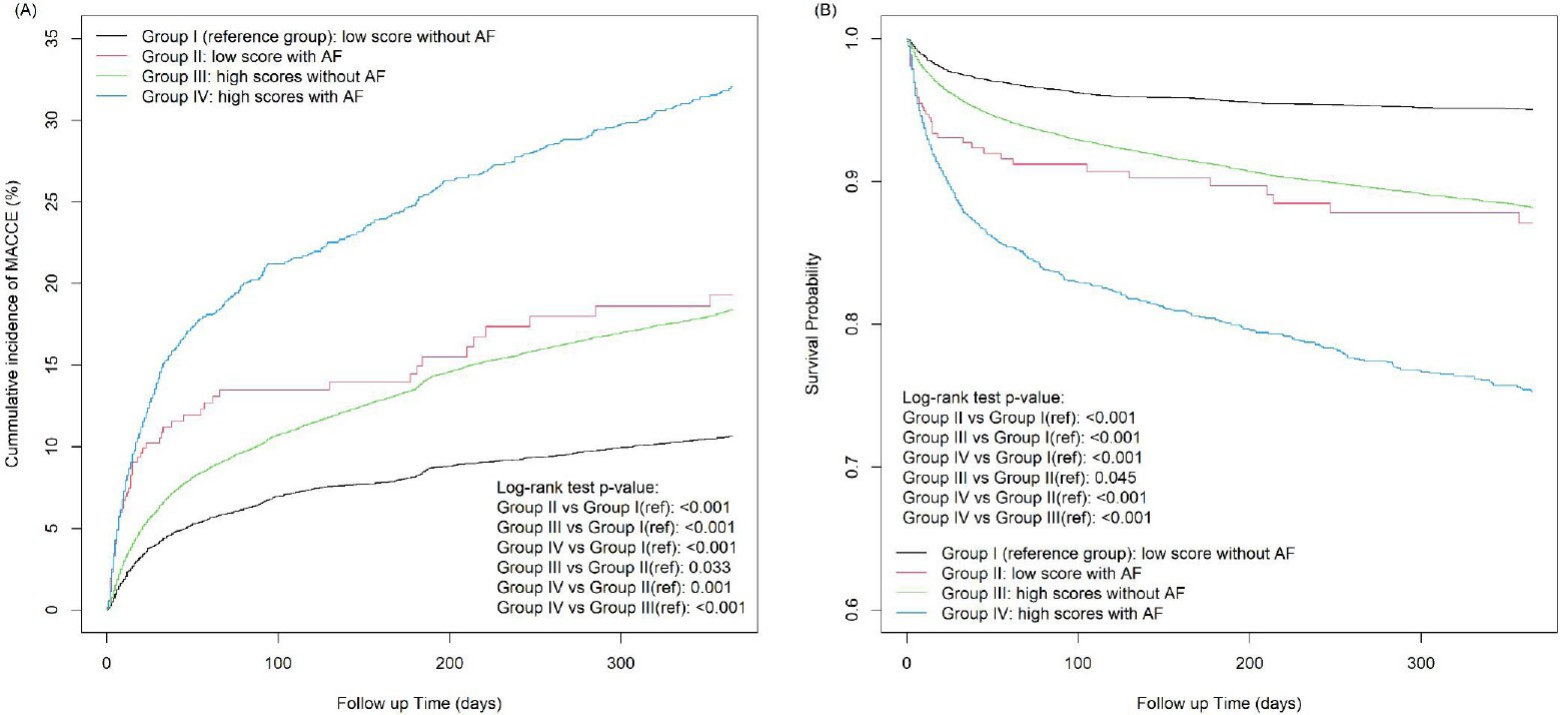
(A) Time-to-Event curves for the major adverse cardiovascular and cerebrovascular events; (B) Kaplan-Meier survival curves for all-cause mortality.

The Kaplan–Meier curves showed that the cumulative incidence of MACCEs was the highest in Group IV (32.1%) and the lowest in Group I (10.6%; Fig 2A). Similar cumulative incidences were observed in Group II (19.3%) and Group III (18.4%). Among patients with AF, Group IV had a higher incidence of MACCEs than did Group II (crude HR = 1.57; p-value = 0.001). Among patients with high CHA2DS2-VASc scores, Group IV had a higher incidence of MACCEs than did Group III (crude HR = 2.06; p-value < 0.001).

Fig 2B shows that the event-free survival rate for all-cause mortality was the lowest in Group IV (75.3%) and the highest in Group I (95.0%). Similar survival probabilities were observed in Group II (87.1%) and Group III (88.1%). Among patients with AF, Group IV had a higher probability of mortality than did Group II (crude HR = 1.81; p-value < 0.001). Among patients with high CHA2DS2-VASc scores, Group IV had a higher incidence of MACCEs than Group III did (crude HR = 2.47; p-value < 0.001).

These results demonstrated that both a high CHA2DS2-VASc score and AF are independent predictors and have an additional effect of unfavorable 1-year outcomes after AIS.

### Individual components of the CHA2DS2-VASc score

**Fig 3:**
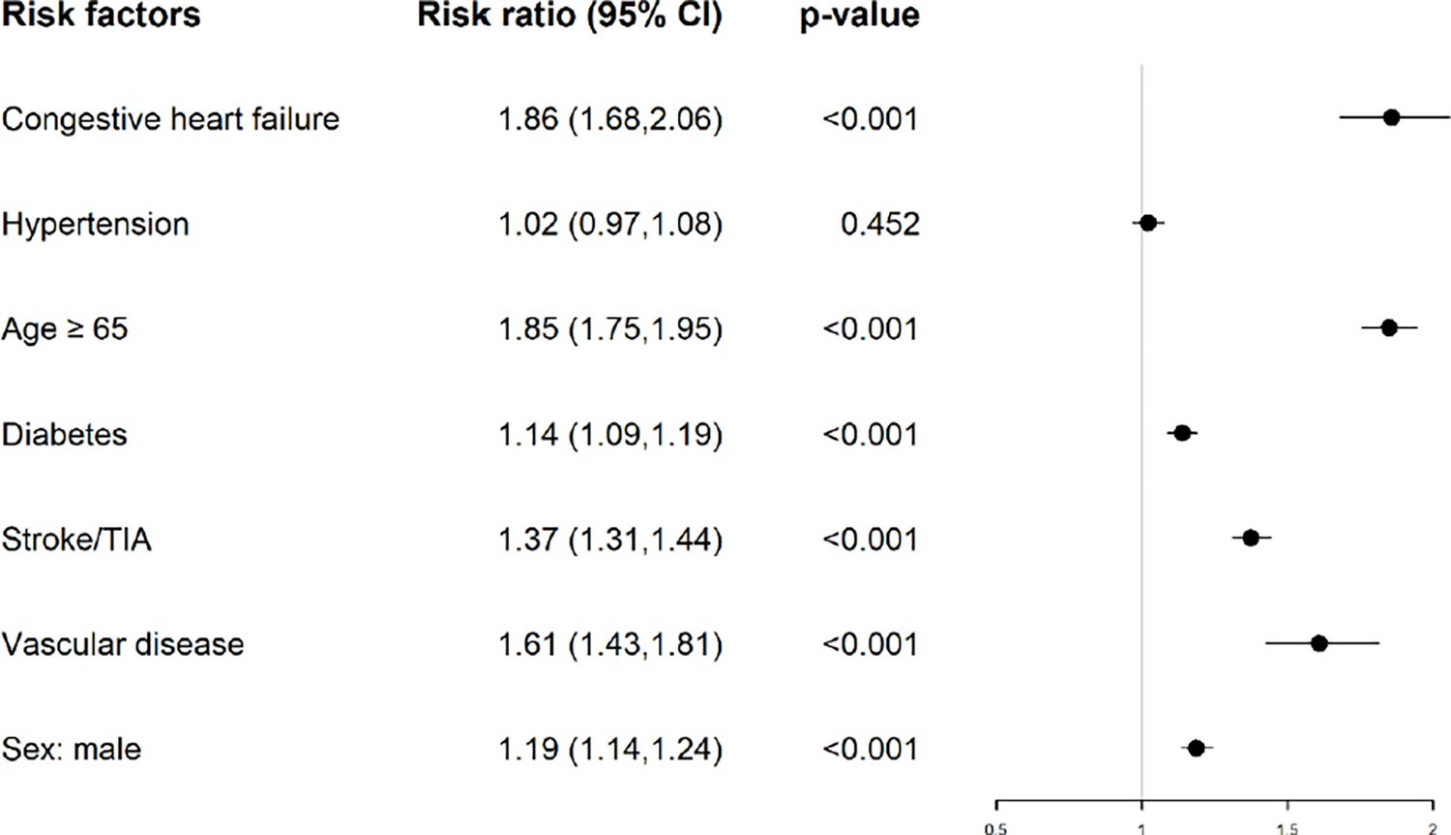
1 year MACCE associated with individual risk factor of CHA2DS2-VASc score.

In the investigation of the effect of individual risk factors in the CHA2DS2-VASc score (Fig 3), age > 65 years, male sex, and a previous history of congestive heart failure, DM, previous stroke/TIA, and vascular disease significantly increased the risk ratio of MACCEs. Patients with a previous history of hypertension had an increasing MACCE trend that did not reach statistical significance, this results may possibly explained by anti-hypertensive agents administration

## Discussion

This study was based on the TSR [15], a nationwide, large-scale stroke registry with rigorous control of entry data, to compare 1-year MACCEs after AIS. The data demonstrated that both a high CHA2DS2-VASc score and presence of AF can independently predict unfavorable clinical outcomes. From these results, we can conclude that the CHA2DS2-VASc score at admission is an effective tool for AIS patients with or without AF. Because this scoring system is common and simple, it can be used as a risk-stratification system after AIS that can help physicians identify patients with poor prognoses.

Previous studies have reported AF to be an independent outcome predictor of AIS caused by potential cardiac embolism and large infarct size [3, 16]. Some studies have addressed the effect of the CHA2DS2-VASc score on clinical outcomes after AIS. Tu et al. [13] conducted a study that included 6,612 patients with AIS, of whom 26.5% had AF. The study found that a CHA2DS2-VASc score of ≥2 was associated with higher mortality and more serious adverse cardiac events (acute coronary syndrome, symptomatic heart failure, cardiopulmonary arrest, life-threatening arrhythmia, and cardiac death) 3 months after admission. Ntaios et al. [17] also reported that prestroke CHA2DS2-VASc scores predict long-term stroke outcomes (mortality, stroke recurrence, and cardiovascular events) in 1,756 patients without AF. However, Yang et al. [18] reported that the CHA2DS2-VASc score cannot predict both mortality and re-stroke in patients with lacunar stroke without AF. In summary, whether the CHA2DS2-VASc score and AF are both valuable and independent clinical outcome predictors after AIS remains uncertain.

In 2018, Su et al. [14] studied 1,494 AIS patients (13% AF) and demonstrated that higher CHA2DS2-VASc score was associated with higher re-stroke and mortality rates irrespective of the presence of AF after the mean follow-up time of 37.5 months. In the present study, AF remained a strong outcome predictor, but significantly higher CHA2DS2-VASc scores were detected in patients with AF. The authors considered this to be a possibly major reason for poorer outcomes in ischemic stroke patients with AF. However, the results couldn’t clarify the independent predictor value of AF in AIS due to insufficient sample size. In contrast, the patient numbers of our large-scale cohort study allowed us to analyze this issue. After adjustment for BMI and all confounding risk factors in the CHA2DS2-VASc score, patients with AF still had significantly higher odds of 1-year MACCEs and all-cause mortality than those without AF. These results provide stronger evidence of the independent predictive value of the AF in patients with AIS. In addition, inconsistent with previous studies [14, 18], AF was associated with the incidence of 1-year re-stroke despite the common use of anticoagulation agents. More importantly, our results reveal that high CHA2DS2-VASc scores were also significantly associated with higher odds of 1-year MACCEs and all-cause mortality compared with low CHA2DS2-VASc scores.

Our study included a total of 7,127 MACCEs and 4559 mortality events in 62,227 patients with AIS. We separately analyzed the predictive effectiveness of AF and CHA2DS2-VASc scores after adjusting for confounding factors. The results reveal that both had strong, independent predictive value for MACCEs and all-cause mortality. The cumulative 1-year incidence of MACCEs and the survival curve provided clearer evidence that high CHA2DS2-VASc scores and AF have an added effect of unfavorable outcomes after AIS.

The stroke types that divided in TSR include large artery atherosclerosis, small vessel occlusion and cardioembolism. The patient numbers of these three types are not significantly different between high and low CHA2DS2-VASc score groups. Previous study demonstrated stroke severity measured by the baseline National Institutes of Health Stroke Scale (NIHSS) is also a strong predictor of stroke outcome. Comparing to NIHSS, CHA2DS2-VASc score is well-known and easier to calculate. We compared the prediction model with CHA2DS2-VASc score and NHISS by area under curve (AUC) of receiver operating characteristic curve (ROC). There is no significance difference between two AUC for predicting MACCE (S1 Fig).

Currently, no study has addressed the effect of an individual component of the CHA2DS2-VASc score on clinical outcomes after AIS. In 2010, the INTERSTROKE study [19] reported history of hypertension and DM as significant risk factors in 2,337 patients with AIS. In 2017, Tang et al. [20] reported that low pulse pressure after AIS is associated with unfavorable outcomes based on TSR data. In our large-scale cohort study, the patient number allowed us to evaluate the individual risk factors of the CHA2DS2-VASc score. In addition to age, a history of congestive heart failure, DM, stroke/TIA, and vascular disease were all associated with unfavorable outcomes. Regarding sex, Nielsen et al. [21] reported that female sex is a risk modifier but not a risk factor for stroke in patients with AF. Similarly, our study revealed that female gender was also not a predictor of 1-year MACCEs. According to both the INTERSTROKE study [19] and our results, DM is both a risk factor and an outcome predictor of AIS.

Our study had several potential limitations: (1) Our study design was observational and not a randomized controlled trial. Additional adequately powered prospective clinical trials with larger sample size are necessary to confirm our findings. (2) The registry did not record whether patients performed the 24-h Holter ECG or not. Even for some patients, paroxysmal AF was not detected on ECG or 24-h Holter ECG during admission, and this was a potential reason for the relatively lower incidence of AF in this study compared with that in previous reports. (3) Some patients with AIS were excluded due to loss to follow-up within 1 year after AIS. (4) Patients at higher score group have more comorbidity, that may have impacts on further events.

## Conclusion

To the best of our knowledge, this is the first study to demonstrate that both the CHA2DS2-VASc score and AF are strong and independent risk predictors of 1-year MACCEs after AIS. Therefore, wider application of the CHA2DS2-VASc score may help improve the holistic clinical assessment of AIS patients with and without AF.

## Supporting information

S1 Fig AUC of different variables to predict 1 year MACCE and mortality. (TIF)

S1 Appendix (DOCX)

S1 File (DOCX)
