# Supplementary figures and images for: CHA2DS2-VASc score as an independent outcome predictor in patients hospitalized with acute ischemic stroke
Source: PLoS One. 2022 Jul 13;17(7):e0270823. doi: 10.1371/journal.pone.0270823 (PMC9278736; doi:10.1371/journal.pone.0270823)

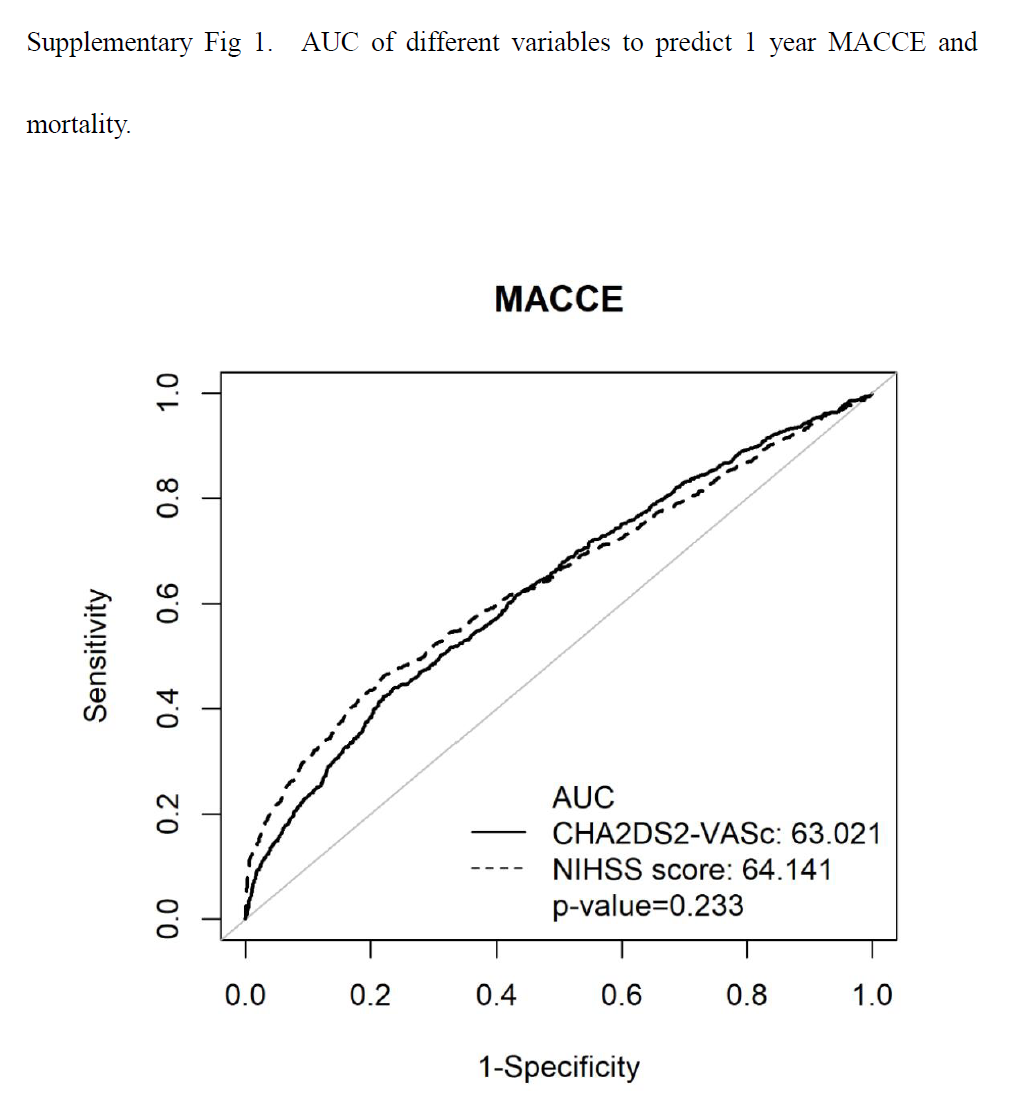

Supplement: S1 Fig — (TIF) [file pone.0270823.s001.tif]
