# Supplementary material for: CHA2DS2-VASc score as an independent outcome predictor in patients hospitalized with acute ischemic stroke
Source: PLoS One. 2022 Jul 13;17(7):e0270823. doi: 10.1371/journal.pone.0270823 (PMC9278736; doi:10.1371/journal.pone.0270823)
Supplement: S1 Appendix — (DOCX) [file pone.0270823.s002.docx]

**List of Taiwan Stroke Registry (TSR) PIs:**

Jiann-Shing Jeng, MD, PhD

Stroke Center and Department of Neurology, National Taiwan University Hospital.

[jsjeng@ntu.edu.tw](mailto:jsjeng@ntu.edu.tw)

Yu Sun, MD, PhD
Department of Neurology, En Chu Kong Hospital, New Taipei City, Taiwan
[sunyu.jj.lu@gmail.com](mailto:sunyu.jj.lu@gmail.com)

Cheng-Yu Wei, MD
Department of Neurology, Chang Bing Show Chwan Memorial Hospital, Changhua County, Taiwan.
Department of Exercise and Health Promotion, College of Education, Chinese Culture University, Taipei, Taiwan.
[yuyu@seed.net.tw](mailto:yuyu@seed.net.tw)

Kai-Cheng Hsu, MD, PhD
Artificial Intelligence Center for Medical Diagnosis, China Medical University, Taichung, Taiwan
School of Medicine, College of Medicine, China Medical University, Taichung, Taiwan
Department of Neurology, China Medical University Hospital, Taichung, Taiwan
[edwardfirst@gmail.com](mailto:edwardfirst@gmail.com)

Po-Yen Yeh

Department of Neurology, St. Martin de Porres Hospital, Chiayi, Taiwan

yeh3652@gmail.com

Wei-Lun Chang
Department of Neurology, Show Chwan Memorial Hospital, Changhua County, Taiwan

a1977611@yahoo.com.tw

Chung Y. Hsu, MD, PhD

Graduate Institute of Biomedical Sciences, China Medical University, Taichung, Taiwan

[hsucy63141@gmail.com](mailto:hsucy63141@gmail.com)

**List of Taiwan Stroke Registry (TSR) Investigators:**

**China Medical University Hospital:** Kai-Cheng Hsu (Principal Investigator),

Chon-Haw Tsai, Wei-Shih Huang, Chung-Ta Lu, Tzung-Chang Tsai, Chun-Hung

Tseng, Kang-Hsu Lin, Woei-Cherng Shyn, Yu-Wan Yang, Yen-Liang Liu, Yuh-

Cherng Guo, Der-Yang Cho, Chun-Chung Chen, Chung-Hsiang Liu

**National Taiwan University Hospital:** Jiann-Shing Jeng (Principal Investigator), Sung-Chun Tang, Li-Kai Tsai, Shin-Joe Yeh

**E-Da Hospital / I-Shou University:**Shih-Pin Hsu (Principal Investigator), Han-Jung Chen, Cheng-Sen Chang, Hung-Chang Kuo, Lian-Hui Lee, Huan-Wen Tsui, Jung-Chi Tsou, Yan-Tang Wang, Yi-Cheng Tai,Kun-Chang Tsai, Yen-Wen Chen, Kan Lu, Po-Chao Liliang, Yu-Tun Tsai, Cheng-Loong Liang, Kuo-Wei Wang, Hao-Kuang Wang, Jui-Sheng Chen,  Po-Yuan Chen, Cien-Leong Chye, Wei-Jie Tzeng, Pei-Hua Wu

**National Cheng Kung University Hospital:** Chih-Hung Chen (Principal

Investigator), Pi-Shan Sung, Han-Chieh Hsieh, Hui-Chen Su

**Shin Kong WHS Memorial Hospital:** Li-Ming Lien (Principal Investigator), Hsu-Ling Yeh, Wei-Hung Chen, Chi-Ieong Lau, Anna Chang, Kuan-Yu Lin

**Kaohsiung Veterans General Hospital:** Ching-Huang Lin (Principal Investigator), Cheng-Chang Yen

**Kaohsiung Medical University Chung**-**Ho Memorial Hospital:** Ruey-Tay Lin

(Principal Investigator), Chun-Hung Chen, Gim-Thean Khor, A-Ching Chao,

Hsiu-Fen Lin, Poyin Huang

**Chi Mei Medical Center:** Huey-Juan Lin (Principal Investigator), Der-Shin Ke,

Chia-Yu Chang, Poh-Shiow Yeh, Kao-Chang Lin, Tain-Junn Cheng, Chih-Ho Chou, Chun-Ming Yang, Hsiu-Chu Shen

**Chung Shan Medical University Hospital:** An-Chih Chen (Principal Investigator), Shih-Jei Tsai, Tsong-Ming Lu, Sheng-Ling Kung, Mei-Ju Lee, Hsi-Hsien Chou

**Show Chwan Memorial Hospital:** Hsin-Yi Chi (Principal Investigator), Chou-Hsiung Pan, Po-Chi Chan, Min-Hsien Hsu, Wei-Lun Chang,Ya-Ying Wu , Zhi-Zang Huang , Hai-Ming Shoung,Yi-Chen Lo, Fu-Hwa Wang

**Cheng Hsin General Hospital:** Ta-Chang Lai (Principal Investigator), Jiu-Haw Yin,

Chung-JenWang, Kai-ChenWang, Li-Mei Chen, Jong-Chyou Denq

**En Chu Kong Hospital:** Yu Sun (Principal Investigator), Chien-Jung Lu, Cheng-Huai Lin, Chieh-Cheng Huang, Chang-Hsiu Liu, Hoi-Fong Chan

**Far Eastern Memorial Hospital:** Siu-Pak Lee (Principal Investigator)

**Kuang Tien General Hospital:** Ming-Hui Sun (Principal Investigator),

Li-Ying Ke

**Taichung Veterans General Hospital:** Po-Lin Chen (Principal Investigator),

Yu-Shan Lee

**Ditmanson Medical Foundation Chia-Yi Christian Hospital**: Sheng-Feng Sung(Principal Investigator), Cheung-Ter Ong, Chi-Shun Wu, Yung-Chu Hsu, Yu-Hsiang Su, Ling-Chien Hung

**Tri-Service General Hospital:** Jiunn-Tay Lee (Principal Investigator), Jiann-Chyun Lin, Yaw-Don Hsu, Jong-Chyou Denq, Giia-Sheun Peng, Chang-Hung Hsu, Chun-Chieh Lin, Che-Hung Yen, Chun-An Cheng, Yueh-Feng Sung, Yuan-Liang Chen, Ming-Tung Lien, Chung-Hsing Chou, Chia-Chen Liu, Fu-Chi Yang, Yi-ChungWu, An-Chen Tso, Yu- Hua Lai, Chun-I Chiang, Chia-Kuang Tsai, Meng-Ta Liu, Ying-Che Lin, Yu-Chuan Hsu

**Cathay General Hospital:** Tsuey-Ru Chiang (Principal Investigator),

Mei-Ching Lee, Pai-Hao Huang, Sian-King Lie, Pin-Wen Liao, Jen-Tse Chen

**Changhua Christian Hospital:** Mu-Chien Sun (Principal Investigator), Tien-Pao Lai, Wei-Liang Chen, Yen-Chun Chen, Ta-Cheng Chen, Wen-Fu Wang, Kwo-Whei Lee, Chen-Shu Chang, Chien-Hsu Lai, Siao-Ya Shih, Chieh-Sen Chuang, Yen-Yu Chen, Chien-Min Chen

**Taipei Tzuchi Hospital**: Shinn-Kuang Lin (Principal Investigator, School of Medicine, Tzuchi University, Hualien, Taiwan), Yu-Chin Su, Cheng-Lun Hsiao, Fu-Yi Yang, Chih-Yang Liu, Han-Lin Chiang.

**Min Sheng General Hospital**: Chun-Yuan Chang (Principal Investigator), I-sheng Lin,Chung-Hsien Chien,Yang-Chuang Chang

**Lin Shin Hospital:** Ping-Kun Chen (Principal Investigator), Pai-Yi Chiu

**National Taiwan University Hospital Yunlin Branch:** Yu-Jen Hsiao (Principal Investigator), Chen-Wen Fang

**Landseed Hospital**: Yu-Wei Chen (Principal Investigator), Kuo-Ying Lee, Yun-Yu Lin, Chen-Hua Li, Hui-Fen Tsai, Chuan-Fa Hsieh, Chih-Dong Yang, Shiumn-Jen Liaw, How-Chin Liao

**Cheng Ching General Hospital**: Shoou-Jeng Yeh (Principal Investigator), Ling-Li

Wu, Liang-Po Hsieh, Yong-Hui Lee, Chung-Wen Chen

**China Medical University Beigang Hospital:** Chih-Shan Hsu(Principal Investigator),Ye-Jian-Jhih, Hao-Yu Zhuang, Yan-Hong Pan, Shin-An Shih

**Taipei Medical University** -**Wan Fang Hospital:** Chin-I Chen (Principal Investigator), Jia-Ying Sung, , Hsing-Yu Weng, Hao-Wen Teng, Jing-Er Lee, Chih-Shan Huang, Shu-Ping Chao

**Taipei Medical University Hospital:** Rey-Yue Yuan (Principal Investigator),

, Jau- Jiuan Sheu, Jia-Ming Yu, Chun-Sum Ho, Ting-Chun Lin

**Kuang Tien General Hospital Dajia Division**: Shih-Chieh Yu(Principal Investigator)

**Changhua Christian Hospital Yunlin Branch:** Jiunn-Rong Chen (Principal

Investigator), Song-Yen Tsai

**Chang Bing Show Chwan Memorial Hospital:** Cheng-Yu Wei (Principal Investigator), Tzu-Hsuan Huang, Chao-Nan Yang, Chao-Hsien Hung, Ian Shih

**Lotung Poh Ai Hospital:**Hung-Pin Tseng (Principal Investigator), Chin-Hsiung Liu, Chun-Liang Lin, Hung-Chih Lin, Pi-Tzu Chen

**Taipei Medical University** - **Shuang Ho Hospital:** Chaur-Jong Hu (Principal Investigator), Nai-Fang Chi, Lung Chan

**Taipei Veterans General Hospital & National Yang-Ming University School of Medicine:**  Chang-Ming Chern (Principal Investigator),   Chun-Jen Lin,  Shuu-Jiun Wang, Li-Chi Hsu,  Wen-Jang Wong, I-Hui Lee, Der-Jen Yen, Ching-Piao Tsai, Shang-Yeong Kwan, Bing-Wen Soong, Shih-Pin Chen, Kwong-Kum Liao, Kung-Ping Lin, Chien Chen, Din-E Shan, Jong-Ling Fuh, Pei-Ning Wang, Yi-Chung Lee, Yu-Hsiang Yu, Hui-Chi  Huang,  Jui-Yao Tsai

**Chi Mei Medical Center, Liouying:** Ming-Hsiu Wu (Principal Investigator),

Shi-Cheng Chen, Szu-Yi Chiang, Chiung-Yao Wang

**Buddhist Dalin Tzu Chi General Hospital:** Ming-Chin Hsu (Principal Investigator)

**St. MARTIN DE PORRES HOSPITAL:** Chien-Chung Chen (Principal Investigator), Po-Yen Yeh, Yu-Tai Tsai, Ko-Yi Wang

**Sin-Lau Hospital, Tainan, the Presbyterian Church in Taiwan**: Tsang-Shan Chen(Principal Investigator)

**Cardinal Tien Hospital:** Ping-Keung Yip (Principal Investigator), Vinchi Wang,

Kaw-ChenWang, Chung-Fen Tsai, Chao-Ching Chen, Chih-Hao Chen, Yi-Chien

Liu, Shao-Yuan Chen, Zi-Hao Zhao, Zhi-Peng Wei

**Yumin Medical Corporation Yumin Hospital**: Shey-Lin Wu(Principal Investigator)

**Kaohsiung Municipal Hsiao-kang Hospital**: Ching-Kuan Liu(Principal Investigator)

**Wei Gong Memorial Hospital:** Ryh-Huei Lin (Principal Investigator), Ching-Hua Chu

**Taipei City Hospital Ren Ai Branch:** Sui-Hing Yan (Principal Investigator),

Yi-Chun Lin, Pei-Yun Chen, Sheng-Huang Hsiao

**National Taiwan University Hospital Hsin-Chu Branch:** Bak-Sau Yip (Principal Investigator), Pei-Chun Tsai,Ping-Chen Chou, Tsam-Ming Kuo, Yi-Chen Lee, Yi-Pin Chiu, Kun-Chang Tsai

**Taichung Hospital Department of Health** : Yi-Sheng Liao (Principal Investigator)

**Tainan Municipal An-Nan Hospital-China Medical University:** Ming-Jun Tsai (Principal Investigator), Hsin-Yi Kao
