# Supplementary material for: CHA2DS2-VASc score as an independent outcome predictor in patients hospitalized with acute ischemic stroke
Source: PLoS One. 2022 Jul 13;17(7):e0270823. doi: 10.1371/journal.pone.0270823 (PMC9278736; doi:10.1371/journal.pone.0270823)
Supplement: S1 File — (DOCX) [file pone.0270823.s003.docx]

**Key Points**

**Question:** Is CHA2DS2-VASc score a valuable risk predictor of clinical outcomes after AIS (acute ischemic stroke)?

**Findings:** In this large-scale prospective cohort study that included 62,227 patients, CHA2DS2-VASc score is strong and independent risk predictor of 1-year MACCEs (a composite of myocardial infarction, re-stroke, or all-cause mortality) after AIS.

**Meaning:** Wider application of the CHA2DS2-VASc score may help improve the holistic clinical assessment of AIS patients with and without AF (atrial fibrillation).
